# From ultrasound images to block based region motion estimation

**DOI:** 10.2349/biij.5.3.e32

**Published:** 2009-07-01

**Authors:** SSS Ranjit, KS Sim, R Besar, CP Tso

**Affiliations:** 1 Faculty of Electronics Engineering and Computer Engineering, Universiti Teknikal Malaysia Melaka, Malacca, Malaysia; 2 Faculty of Engineering and Technology, Multimedia University, Malacca, Malaysia

**Keywords:** Hexagon search, motion estimation, ultrasound images, diamond search, block-matching

## Abstract

By applying a hexagon-diamond search (HDS) method to an ultrasound image, the path of an object is able to be monitored by extracting images into macro-blocks, thereby achieving image redundancy is reduced from one frame to another, and also ascertaining the motion vector within the parameters searched. The HDS algorithm uses six search points to form the six sides of the hexagon pattern, a centre point, and a further four search points to create diamond pattern within the hexagon that clarifies the focus of the subject area.

## INTRODUCTION

The current status quo in ultrasound research lends itself to a process where anticipated motion coordinates are calculated through the identification of objects in motion within a series of images [1]. The incorporation of block-matching into the process allows the prediction of the expected range of motion, by comparing the previous and current image instances in the series [2]. Further extension of these principles occurs through focusing on a specific region of interest within an image, and in this way block-matching can be used to interpret ultrasound images for such purposes as medical heart translation [3].

By combining block-matching processes with optical flow, atrial septal defects have since been able to be diagnosed with great accuracy, simply by identifying the incongruent elements inherent in two consecutive frames [4]. While applying this measurement of difference to ultrasound images, Singh’s formula also incorporated block-matching, and the application of velocity estimation in ultrasound images [5]. Further, block-matching has been used to identify the precise dimensions in 3-D ultrasound images [6], and this ability has led to the use of block-matching to identify fetal development and movement [7].

Due to the fact that compression of video often leads to temporary redundancy, the block-matching algorithm is found to be able to decrease this redundancy in many frameworks, and has enjoyed great favour by users [8]. To identify the precise change in position of the most favourably matched block vis-a-vis the preceding frame, block-based motion estimation mimics the motion vector to the block in the current frame within the search area, by dividing the frames similarly, into equally sized rectangular blocks.

Clearly, the hexagon-diamond search (HDS) technique warrants being applied to ultrasound imaging through video sequences. These video sequences, apart from assisting a diagnosis, will capture the true condition of subjects, and as less memory is utilised to store data, high transmission of data is possible to affect video conferencing.

MATLAB (The Mathworks, Natick, MA) is used to execute these intensive computations, and allows for the dynamic monitoring of motion translation within the video sequence. This allows calculation of the motion vector, which is then incorporated into the process when the challenging area is scrutinised.

## PROPOSED SOLUTION

The combination of the hexagon search pattern and its corresponding diamond search pattern is in essence a process of primary searching followed by verification of primary coordinates to result in a far more precise output.

The focus of the search is within the six search points of the outer hexagon pattern, with a seventh point taking up position at its centre. Around this centre, another four search points form a small diamond pattern within the hexagon pattern, which clarifies the search.

The hexagon is the one shape that addresses the vertical and horizontal block displacement that is found in most video sequences, by adapting its operation to match this directionality.

In order to affect faster block-matching computation speed, the mean absolute difference (MAD) is the criteria of choice that is compared. When the MAD is utilised in this fashion, the peak signal to noise ratio (PSNR) of the target video is reduced, and it enables bleeding edge analysis of block matching algorithms (BMA) [9].

If one is familiar with search methods applied to various parameters and shapes, fast block-matching algorithms are able to successfully analyse the search speed and performance of any given search method [10]. Invariably, 50-90% of motion vectors are within a circle of a two pixel radius, its centre bearing zero motion [9].

The following search method uses two different sub-searches, each of a different shape.

- **Step 1** The six search points that form the hexagonal and the seventh point at its centre undergo verification and comparison with one another. The minimum motion vector will then be identified; if the minimum MAD calculation is to be had at the centre of the hexagon, jump to Step 4.
- **Step 2** If not, the hexagonal search is repeated until the minimum MAD is at the centre of the hexagonal.
- **Step 3** The minimum MAD that is now the centre of the hexagonal now forms the centre of the small diamond.
- **Step 4** The search then jumps to the small diamond search to clarify the minimum motion vector and make comparisons between the four points of the diamond to ascertain the best minimum MAD.
- **Step 5** Once this search has found the minimum MAD in the current block it will move to the next one.

Due to their configuration, the search points above will resolve vertical and horizontal block displacements. It is with respect to this inherent ability that the region of interest will be scrutinised. The use of less frequent search points reduces processing time, and increases the overall performance of the algorithm in when locating the region of interest

## RESULTS AND DISCUSSION

In order to monitor performance of the HDS algorithm, in addition to capturing each frame and calculating its PSNR points, the predicted frame is compared to the reference frame. The resulting displacement is used to reveal the condition of the region of interest.

The following initialisation is implemented:

- MAD block size 16 x 16 pixels
- Search window size 15 x 15 pixels
- Ultrasound video sequence 176 x 144 pixels at 5 frame per second (f.p.s.)

The hexagon pattern with its six points and centre point is used to search until the optimum motion vectors are identified (MAD_0,_ MAD_1,_ MAD_2_ and MAD_3)._ Then the small diamond search pattern clarifies MAD_4_ and verifies that it is the optimal motion vector from comparing the four search points of the small diamond pattern.

The number of PSNR points in the reference video sequence is greater than in the newly constructed video sequence. This is apparently due to the compression of the image [9], but a reduction in PSNR points has the collateral benefit of also offering an image with higher definition.

Adding to better image quality, converse to the frequency of PSNR points, the number of search points increases when the image is compressed to produce the reconstructed video sequence (an increase of 1.22 search points at 5 f.p.s. and an increase of 2.42 search points at 10 f.p.s).

As a result of the HDS algorithm, at 5 f.p.s. the PSNR of the original video sequence undergoes a 29.4% compression, and at 10 f.p.s. it undergoes 27.6% compression. This is conclusive evidence of temporal redundancy being reduced, and the increase in the number of search points makes the exercise of realising of the best matching block in the processed target frame more accurate.

The results of the HDS algorithm in use reveal a small change from original image to the reconstructed image.Figures 4(a), 5(a), 6(a), and 7(a) all represent the original video sequence, and Figures 4(b), 5(b), 6(b) and 7(b) the reconstructed sequence. When the HDS algorithm is applied, the best motion vector for the target image for the fine tuning search comprises of the following respectively: (8,7), (9,9), (8,9) and (8,9). These coordinates are the point at which motion can be monitored with precision; they are the position of the best matching block in the preceding frame. When the frames of the original video sequence are compared to the reconstructed frames, the temporal redundancy is patently obvious. Compression is evident in the number of PSNR points decreasing in the resultant video sequence (Table 1), which shows that compression is taking place and removes the temporal redundancy. This vector smoothing technique is a result of the fact that the motion vector has a more pronounced impact on the compression ratio.

**Figure 1:**
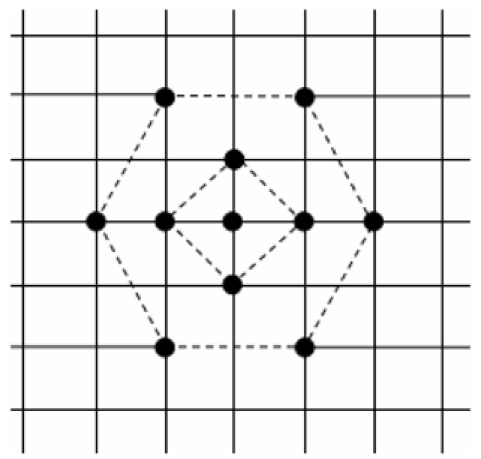
A schematic of hexagon-diamond search algorithm.

**Figure 2:**
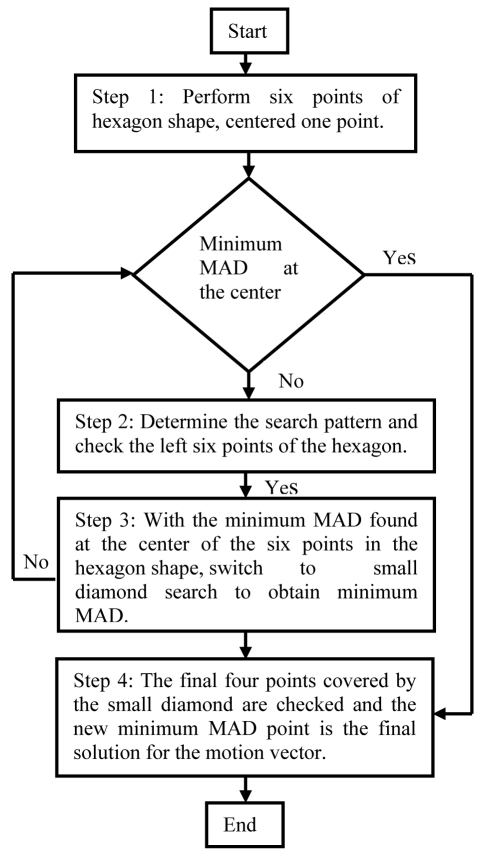
HDS method flowchart.

**Figure 3:**
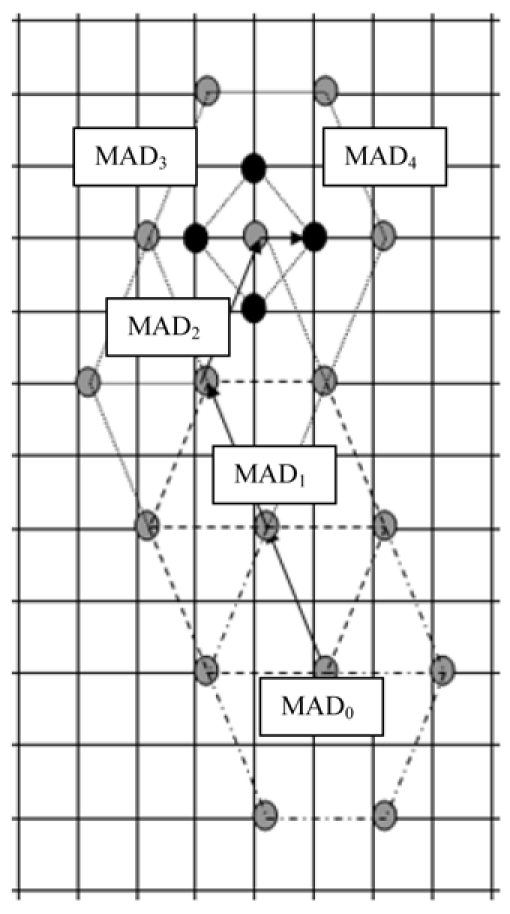
Motion vector search method.

**Figure 4:**
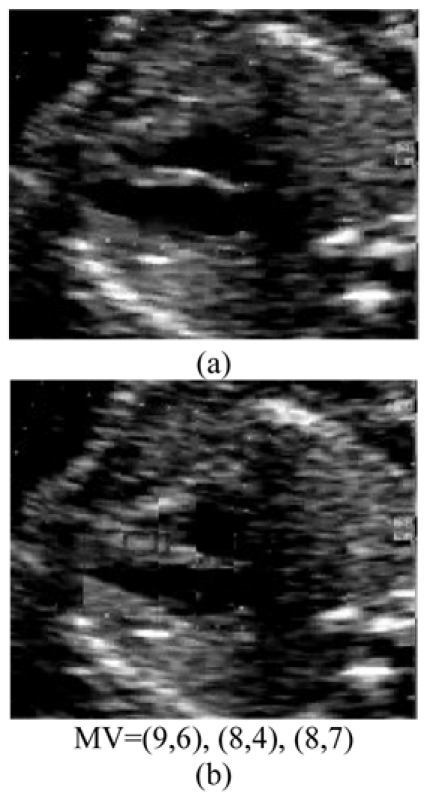
(a) Original ultrasound image and (b) Predicted ultrasound image

**Figure 5:**
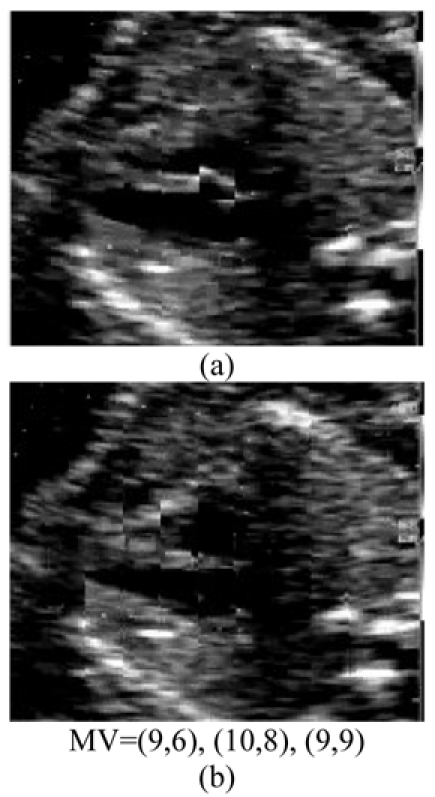
(a) Re-processed original ultrasound image and (b) Predicted ultrasound image

**Figure 6:**
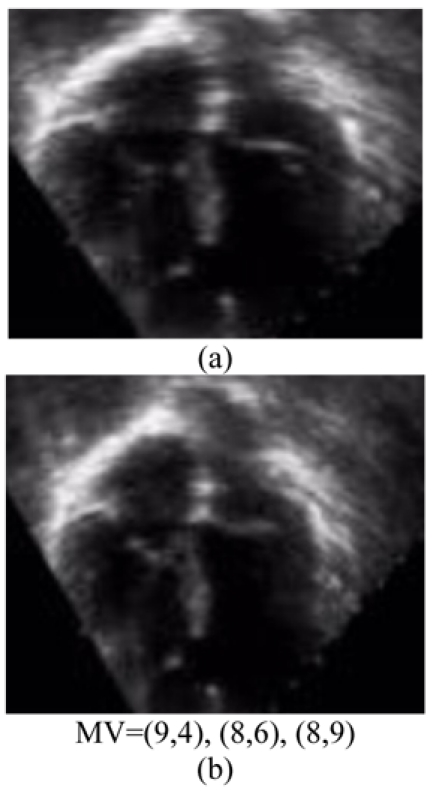
(a) Original ultrasound image and (b) Predicted ultrasound image

**Figure 7:**
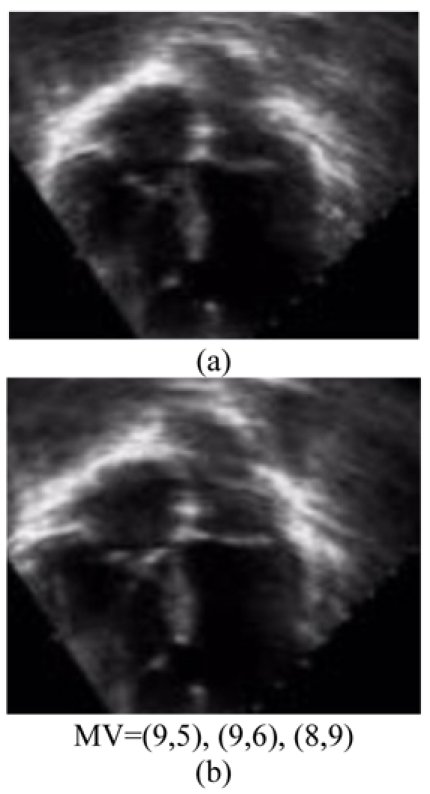
(a) Re-processed original ultrasound image and (b) Predicted ultrasound image

**Table 1:** Algorithm output at 5 and 10 f.p.s

	**5 f.p.s**		**10 f.p.s**	
	**Original video**	**Re-processed video**	**Original video**	**Re-processed video**
PSNR points (dB)	26.41	18.66	24.44	17.69
Average search point	13.56	15.78	12.44	14.86
Average time (sec)	1.91	1.91	2.52	2.52

As in Figures 4(b), 6(b), and 7(b), when the motion vector magnitudes are increasing in order, the change from the reference point will not be constant. The best matching point in both these instances is on the vertical plane. The motion vector magnitude in Figure 5(b), however, can be read to be getting larger or smaller, and the coordinates reveal that the best matching point is on the horizontal plane.

## CONCLUSION

The hexagon-diamond search algorithm has the ability to optimise ultrasound video sequence block-matching for motion estimation. This functionality is indispensable in diagnostics, and is portable enough to be applied to image compression and reconfiguration of ultrasound video sequences. In this manner, bleeding edge transmission can take place even with conduits of meagre capacity.
